# Quantifying the demand for central carbon metabolism precursors for biosynthesis in proliferating cells

**DOI:** 10.1186/2049-3002-2-S1-P35

**Published:** 2014-05-28

**Authors:** Mark Keibler, Thomas Wasylenko, Joanne Kelleher, Gregory Stephanopoulos

**Affiliations:** 1 Department of Chemical Engineering, Massachusetts Institute of Technology, Cambridge, MA, USA

## 

Metabolic flux comprises the ultimate phenotypic readout of cell physiology, and it shifts toward anabolic or catabolic regimes in conjunction with the proliferative state of the cell. Many parallels exist between the metabolic profiles of normal dividing cells and cancer cells, for which uncontrolled proliferation is a hallmark. Much effort in the field of cancer metabolism has been directed toward identifying these similarities to target how tumors use biochemical resources for growth and survival.

Many studies have approached this question by experimentally identifying major metabolic changes that become induced in response to expression of oncogenes or inactivation of tumor suppressor genes; however, in these circumstances, the generality of these changes beyond the system under consideration may be unknown. Other avenues have employed genome-scale models of tumor metabolism to determine enzymes through which flux is upregulated when biomass production is maximized, but the size of these networks limits routine analysis.

All major components of macromolecules may be derived from a small set of central metabolites that originate from well-known metabolic pathways, and as a result, it is possible to determine the rates at which these precursors must be consumed to maintain a particular growth rate. We have therefore employed a method to reduce experimentally measured biomass components to this set of precursor metabolites to better understand the effects of growth on metabolism in mammalian cells. Our strategy enables generalization of biosynthetic demands in a manner that is not specific for any particular oncogenic genotype while using a limited biochemical currency that enables elementary calculations and predictions.

